# Phenotypic Characterization of Carcass Traits, Organ Weights, Reproductive Organ Measurements and Tissue Chemical Composition in Three Sheep Breeds

**DOI:** 10.3390/vetsci13040379

**Published:** 2026-04-14

**Authors:** Ahmed A. Saleh, Nasir A. Ibrahim

**Affiliations:** 1 Animal and Fish Production Department, Faculty of Agriculture (*Al-Shatby*), Alexandria University, Alexandria 11865, Egypt; 2 Department of Biology, College of Science, Imam Mohammad Ibn Saud Islamic University (IMSIU), Riyadh 11623, Saudi Arabia

**Keywords:** sheep, carcass traits, organ weights, reproductive organs, meat composition

## Abstract

This study aimed to characterize carcass traits, organ weights, reproductive organ measurements, meat chemical composition, and gastrointestinal tract characteristics in Barki (BAR), Rahmani (RAH), and their crossbred BAR × RAH sheep. A total of 30 adult sheep, consisting of 10 animals from each breed group, (5 males and 5 females per group), aged approximately 36.5 ± 0.75 months, were randomly selected from the flock raised under uniform management systems in northern Egypt. The animals were subjected to comprehensive phenotypic evaluation following slaughter. The crossbred BAR × RAH animals were found to be superior for all weight-related traits since they had the highest mean values for cold carcass weight (30.12 ± 2.43 kg), final live body weight (59.46 ± 1.28 kg), slaughter weight (58.27 ± 2.19 kg), as well as trimmed meat weight (23.70 ± 1.35 kg), all of which were significantly higher than those of both purebred breeds (*p* < 0.001). BAR breed had the lowest values in the weight-related traits but the most favorable ratio of lean-to-fat tissue (4.12 ± 0.92) and the highest content of ash in the meat (1.00 ± 0.05%). Pronounced breed differences were observed in the measurements of the reproductive organs. Chemical analysis of the meat revealed that the meat of the crossbred animals contained the highest amount of moisture (74.66 ± 1.99%) and crude protein (21.65 ± 0.57%). The results of the GI tract revealed that the crossbred animals had the longest intestines (41.17 ± 2.18 m). The pH of the animals also showed breed-specific characteristics. Crossbreeding BAR and RAH improved growth, carcass and reproductive traits, supporting its use in breeding programs.

## Simple Summary

This study was done on Barki sheep, which can survive under harsh weather conditions but grow slowly, Rahmani sheep, which grow fast but deposit more fat, and the crossbred offspring of both breeds, with the objective of finding the most suitable for meat production in Egypt. The study was done on 30 adult sheep, focusing on body weight, carcass weight, organ size, reproductive organ size, fat distribution and meat quality. The crossbred offspring inherited the good characteristics of both parents, thus performing better in terms of growth rate, lean meat percentage, reproductive organ size, meat quality, protein and fat composition compared to the pure breeds. The pure Rahmani sheep, particularly males, exhibited more fat deposition, while Barki sheep, despite being smaller, had the leanest meat with the best muscle-to-fat ratio. This study is highly recommended for farmers, as it shows that crossbreeding Barki and Rahmani sheep is the way forward for the increased demand for mutton products in Egypt while promoting the conservation of indigenous livestock genetic resources.

## 1. Introduction

Sheep production plays an important role in the agricultural sector of Egypt, and this sector makes significant contributions to the country’s meat production and the use of marginal land [1,2,3]. The sheep population in Egypt is made up of indigenous breeds that have developed unique adaptive characteristics and production potentials due to the diverse agro-ecological environments [4]. Of these indigenous breeds, Barki (BAR) and Rahmani (RAH) sheep represent two unique genetic resources traditionally farmed by smallholder farmers and pastoral communities in different geographic regions of Egypt [5,6]. The total sheep population in Egypt is approximately 5.49 million heads, with the three major indigenous breeds, BAR, RAH and Ossimi (OSI), accounting for about 65% of the national flock. BAR sheep represent roughly 11%, and RAH with OSI together constitute approximately 35% [3,5].

BAR sheep, named after the city of Barka in Libya, inhabit the northwestern coastal zone of Egypt, stretching from Alexandria to the Libyan border. BAR sheep have demonstrated impressive adaptability to the extreme desert environment, which is usually subjected to extreme solar radiation, precipitation fluctuations, high temperatures and feed scarcity [4]. BAR sheep are usually raised under a system of extensive management systems where they graze on rangelands and salt-tolerant shrubs dominating the pastoral vegetation. BAR sheep exhibit a number of characteristics such as their fat-tailed conformation, coarse wool covering, fertility under stressful conditions, and adequate mothering ability [4]. Adult BAR ewes typically weigh 35–50 kg and rams 50–60 kg; they are considered non-seasonal breeders with moderate fertility, achieving lambing rates of 61–100% under favorable conditions [4,5]. However, the growth rate of BAR sheep is considered to be moderate in comparison to the other Egyptian sheep breeds. Moreover, the body weights of BAR sheep at marketing age and the carcass yields are less than those of the other Egyptian sheep breeds [4,5,7,8]. Abousoliman et al. [9] reported the significant association of the polymorphism in *LEP* gene with the weaning weight and average daily gain in the pre-weaning period in BAR sheep lambs.

On the contrary, RAH sheep is considered one of the most productive native breeds in Egypt, mostly found in the Nile Delta region, specifically in the areas of the Kafr El-Sheikh governorate and its surroundings [10,11,12]. This breed is known for its superior growth rate and body weights in comparison to other native Egyptian sheep breeds such as BAR and Ossimi [10,13]. Adult RAH ewes weigh 55–65 kg, and rams weigh 65–85 kg; they are also non-seasonal breeders with lambing rates of 100–120% under intensive management [10,14]. Recently, Rabaa et al. [5] proved that the growth performance of RAH sheep lambs is superior in intensive production systems, although BAR sheep lambs had similar body weights to RAH sheep in specific developmental periods. RAH sheep have a phenotypic appearance characterized by a brown or tan coat color. They also have a moderately sized fat tail. Reproductive performance is generally good but not as prolific as that of some maternal breeds [14,15].

On the other hand, crossbreeding is an effective strategy to improve growth, carcass, meat, and reproductive traits by exploiting heterosis, as demonstrated in various sheep breeds [16,17,18,19]. Crossbreeding BAR with RAH offers a promising strategy to combine the adaptive hardiness of BAR with the higher growth potential of RAH, which is particularly relevant for meeting the rising demand for mutton in Egypt [5]. The success of such programs, however, relies on detailed knowledge of phenotypic traits, including carcass composition, organ development, reproductive characteristics, meat quality, and gastrointestinal physiology [20,21,22,23].

Carcass traits such as cold carcass weight, dressing percentage, primal cut weights, tissue composition, and fat deposition are key determinants of production efficiency and meat quality [16,17,24,25]. Fat distribution particularly in depots like the fat tail, perinephric, and omental fat affects carcass leanness and economic value, whereas intramuscular fat contributes to palatability and flavor [16,26]. The rib eye area (REA) serves as an indicator of muscular development and lean yield [16]. Organ weights (heart, liver, kidneys, lungs and spleen) reflect genetic influences on visceral development and adaptation [27,28,29], and gastrointestinal tract (GIT) characteristics (stomach and intestinal weights, and pH) have been shown to vary among breeds [27]. Reproductive organ dimensions (testes, ovaries, uterus and oviducts) provide insights into breed differences in fertility [30,31].

The chemical composition of meat has a significant impact on the nutritional and sensory properties of meat, which in turn affects the acceptance of meat by consumers [32]. Moisture content, crude protein, intramuscular fat content (total lipids), and ash content are the major chemical composition of meat, and the proportion of these composition in meat is the key to the determination of the quality characteristics of meat [16]. Liang et al. [32] reported breed differences in amino acid composition and protein characteristics among three Xinjiang lamb breeds (Duolang, Hetian and Qira Black). In addition, protein content is of great importance from a nutritional perspective, and fat content influences flavor, tenderness and juiciness of the final product [33,34,35]. Collagen content, as part of the connective tissue of animals, influences the tenderness of final meat products, and lower concentrations of collagen are related to more tender meat products [32]. Tibaoui et al. [36] indicated that dietary treatments may affect the physicochemical properties of meat, such as fatty acid composition and stability of meat and meat products. Baseline breed differences are of great importance in understanding differences in meat composition. Qiao et al. [16] found that crossbred lamb muscles contained higher concentrations of polyunsaturated fatty acids, n-6 PUFA, and PUFA/SFA ratio than purebred Tibetan sheep, and higher concentrations of glutamic acid and methionine were found in crossbred lamb muscles than purebred Tibetan sheep.

GIT characteristics such as organ weights, tissue masses, intestinal length and pH values in the GIT have not received much attention in studies comparing different breeds, though these parameters play an important role in the efficiency of the digestive process and nutrient absorption [27]. Yıldırım et al. [27] showed significant breed variation in the GIT characteristics among six Turkish sheep breeds. Also, breed differences in ruminal activity have been reported by Ranilla et al. [37], who observed that Churra sheep had higher in situ degradability of forages than Merino sheep, possibly due to the ruminal environment and the activity of microbes. It is widely understood that ruminal pH influences both microbial composition and the production of volatile fatty acids (VFAs), which in turn shapes how efficiently nutrients are extracted from feed [27]. Beyond the rumen, pH conditions in the small intestine, particularly the jejunum and the hindgut (caecum), have also been shown to impact digestive enzyme activity and the make-up of microbial populations in those compartments [38,39]. Work by Ding et al. [40] illustrated this well: they documented clear differences in both pH levels and digestive enzyme activities between Small-tailed Han sheep and Tan sheep, with significant variation across the duodenum, jejunum, and ileum of the two breeds.

The present study was designed to test the hypothesis that crossbreeding between BAR and RAH would produce offspring with improved growth performance, carcass yield and reproductive development compared to the purebreds, while retaining favorable meat quality attributes. These three sheep breeds were selected because BAR represents a hardy, well-adapted desert breed with lean meat but moderate growth; RAH represents a highly productive, fast-growing breed with higher fat deposition; and their crossbred (BAR × RAH) offers the opportunity to evaluate heterosis for traits of economic importance. Accordingly, this study provides a detailed phenotypic description of carcass traits, organ weights, reproductive organ dimensions, and tissue chemical composition of BAR, RAH, and BAR × RAH sheep, supporting informed decision-making in the development of sheep breeding programs and the sustainable utilization of Egyptian indigenous sheep genetic resources for meat production.

## 2. Materials and Methods

### 2.1. Ethical Statement

The Guide for the Care and Use of Agricultural Animals in Research and Teaching (FASS, 2010) was strictly followed in all experimental protocols and procedures. The Animal Care and Use Committee of the Faculty of Agriculture at Alexandria University in Egypt granted ethical clearance for the study protocol (Approval date: 20 September 2022, Approval code: AU082209203103).

### 2.2. Experimental Animals and Management

This study was carried out within the context of a broader research project whose objective was to identify the characteristics of the production attributes of various breeds of sheep in the northern part of Egypt. The original breed stock was collected from two different geographical areas. These areas are the El-Hammam district of the Matrouh Province, which is located at a latitude of 30.833132° N and a longitude of 29.397580° E, and Sakha city, which is located at a latitude of 31.090560° N and a longitude of 30.943543° E.

**Figure 1:**
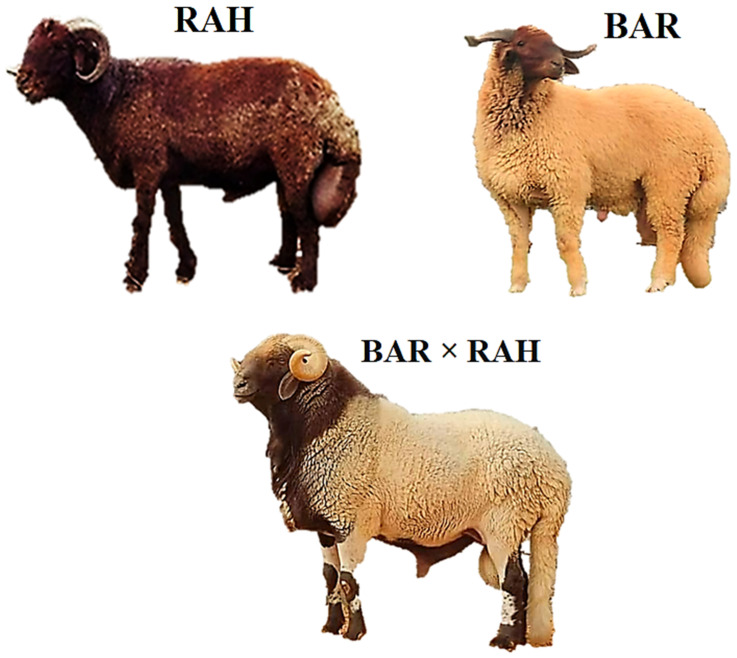
Studied sheep breeds used in the present study; Barki (BAR), Rahmani (RAH), Rahmani × Barki cross (RAH × BAR).

For the investigation of phenotypic characterization presented in this work, a total of 30 adult sheep from three sheep breeds that represent the most important genetic types of interest were selected. These breeds are Barki (BAR; 10 animals, 5 males/5 females), Rahmani (RAH; 10 animals, 5 males/5 females), and their crossbred (BAR × RAH; 10 animals, 5 males/5 females) (Figure 1). The sheep were randomly selected from larger contemporary populations; BAR (*n* = 331), RAH (*n* = 188) and BAR × RAH (*n* = 412), which were part of the parent study. The sample size (10 per breed group) was determined based on the availability of adult animals meeting the inclusion criteria and was considered adequate for detecting significant breed and sex differences, consistent with previous phenotypic characterization studies in sheep. All the sheep selected for the phenotypic characterization were born within a narrow time frame of approximately 2–3 weeks and had a mean age of 36.5 ± 0.75 months at the beginning of the study. All animals originated from single lambing, as multiple births are uncommon in these breeds under the management conditions represented. Only healthy, non-pregnant (females) and non-lactating animals with a body condition score of 3.0–3.5 (on a 5-point scale) were included to minimize variation due to reproductive or nutritional status. Throughout the period of the experiment, the sheep were kept on a semi-intensive system of rearing in well-ventilated and well-lit concrete barns that allow the sheep to move around. Nutrition consisted of a total mixed ration offered twice daily (08:00 and 16:00) at a level to meet maintenance requirements, composed of 40% concentrate and 60% roughage on a dry matter basis. The concentrate was formulated from ground yellow corn (60%), soybean meal (25%), wheat bran (10%), limestone (1.5%), salt (0.5%) and a vitamin–mineral premix (3%), providing on a dry matter basis: crude protein 14.0%, ether extract 3.2%, crude fiber 6.5% and metabolizable energy 2.50 Mcal/kg. The roughage was berseem hay (*Trifolium alexandrinum*) harvested at early bloom, with a chemical composition of crude protein 16.0%, neutral detergent fiber 45.0%, acid detergent fiber 32.0%, and ash 9.5% on a dry matter basis. Fresh water was available ad libitum. The environmental and nutritional conditions were identical for studied sheep breeds. El-Hammam city, northern Egypt, where the study was conducted, is characterized by a Mediterranean coastal climate with hot, dry summers (average maximum temperature 32–35 °C) and mild, moderately wet winters (average minimum temperature 8–12 °C); annual rainfall ranges from 100 to 150 mm, occurring mainly in winter.

### 2.3. Slaughter Protocol

Firstly, all texted animals were weighed individually utilizing a calibrated scale. The animals then underwent a fasting process for 18 h, with ad libitum access to water [41]. A second body weight measurement was recorded immediately before slaughter to determine the fasted live weight. The slaughter procedure followed standard commercial practices employed in Egyptian abattoirs.

### 2.4. Post-Slaughter Processing and Sample Collection

Subsequent to bleeding, the head was removed along the atlanto-occipital joint, and the feet were removed along the carpal and tarsal joints. The process of dressing the carcass began with partial skinning while the animal was in dorsal recumbency, followed by hanging the animal by the hind limbs for completion of the skinning process. All carcass and non-carcass parts were weighed immediately after collection. For heavier components (carcass sides, primal cuts, fat depots, and gastrointestinal tract segments), a floor scale (Model 2888, Mettler-Toledo, Columbus, OH, USA) was used; weight was recorded in kilograms. Organs and non-carcass components were weighed on a precision electronic balance (Model BL3200H, Shimadzu Corporation, Kyoto, Japan), and weight was recorded in grams.

### 2.5. Collection of Carcass and Non-Carcass Components

Non-carcass components were also collected and their weights determined. We collected the non-carcass parts and grouped them as: respiratory (lungs and trachea), circulatory (heart), digestive (full and empty tract, liver, spleen and pancreas), and others (head, skin and feet). To get the weight of the gut contents, we subtracted the weight of the empty digestive tract from the full one. The empty live weight of the animals was recorded.

### 2.6. Carcass Dissection and Physical Assessment

Carcasses were chilled at 4 °C for 24 h, after which the left side of each carcass was utilized for detailed physical dissection following established protocols by Sen et al. [42] and Santos et al. [43]. The carcass side was separated into individual primal cuts, and the *Longissimus dorsi* muscle area (REA) was measured at the interface between the 10th and 11th ribs using planimetry.

Systematic manual dissection of each primal cut was performed to separate the tissues into three fundamental components: lean tissue (trimmed meat), bone, and dissected fat. Each component was weighed separately to determine absolute weights (kg) and proportional contributions to the side weight (%). Several calculated indices were derived from these measurements, including the lean-to-fat ratio, lean-to-bone ratio, and the ratio of cold carcass weight to fasted live weight (expressed as a percentage).

### 2.7. Carcass Linear Measurements

Linear measurements of the carcass were obtained with a flexible measuring tape and a digital caliper (Absolute Digimatic Caliper, Model CD 15APX, Mitutoyo Corporation, Kawasaki, Japan). These measurements included the length of the carcass, round length and circumference, loin length, and width of the carcass at the 3rd and 7th ribs. All measurements were recorded in centimeters by the same trained individual to minimize operator variability.

### 2.8. Organ Weights and Reproductive Tract Measurements

After complete evisceration, each internal organ was carefully dissected from the adhering connective tissue and fat, blotted on paper towels, and weighed on a precision electronic balance (Model BL3200H, Shimadzu Corporation, Kyoto, Japan); weight was recorded in grams. The organs weighed included the heart, liver, kidneys, lungs with trachea, and spleen.

The reproductive organs were excised and processed separately. In males, both testes were dissected free of the scrotal sac and epididymis, and their combined weight was recorded. In females, the entire reproductive tract was collected, and the ovaries, ovarian tubes, and uterus were separated, each of which was weighed individually. In addition, the lengths of the ovarian tubes and uterine horns were measured in centimeters using a digital caliper.

### 2.9. Gastrointestinal Tract Measurements

GIT was completely removed and separated into its constituent parts. The rumen was isolated, and the contents were sampled for pH determination by inserting a digital pH meter (Model HI98129, Hanna Instruments, Woonsocket, RI, USA) into the contents of the rumen. Similar measurements of pH values were taken from the jejunum and caecum by making small incisions and inserting the same pH meter into the contents of the intestine.

After obtaining the pH values of the content of the rumen, the entire stomach and intestines were weighed. The content of the stomach and intestines was removed. The entire stomach and intestines were rinsed with physiological saline solution. After that, the stomach and intestines were blotted to remove the moisture of the content of the stomach and intestines. It was done in order to weigh the stomach and intestines after removing the content. The entire small and large intestines combined were measured by stretching the intestines along a measuring tape without any tension.

### 2.10. Fat Depot Measurements

Each fat depot was dissected and weighed according to the methodologies proposed by Ekiz et al. [44] and Kirton et al. [45]. The fat depots collected were as follows: (a) Heart fat (pericardial fat): fat tissue around the heart. (b) Kidney fat (perinephric and pelvic fat): fat tissue around the kidneys and the pelvic canal. (c) Gut fat (omental and mesenteric fat): fat tissue around the gut. (d) Fat tail: the entire fat tail was removed and weighed separately.

Each fat depot was weighed separately using a precision balance. The weight of each fat depot was noted in grams, except for the fat tail, which was noted in kilograms. The total fat content of each animal was determined by adding the weights of each fat depot.

### 2.11. Blood Volume Determination

During exsanguination, the total blood volume was collected in graduated containers and measured directly. The volume of blood was recorded in liters for each animal.

### 2.12. Chemical Composition Analysis of Meat

The chemical composition of the meat was determined using samples collected from the Longissimus dorsi muscle at the level of the 10th rib, following the standard procedures outlined by the Association of Official Analytical Chemists [46] and Madruga et al. [47]. Approximately 100 g of tissue was collected from the left side of the carcasses, trimmed to remove all visible epimysial connective tissue and fat, leaving only lean tissue.

The trimmed tissue was frozen at −80 °C, then freeze-dried (Alpha 1-4 LDplus, Martin Christ Gefriertrocknungsanlagen GmbH, Osterode am Harz, Germany) until constant weight. Dried samples were ground using a mill (ZM 200, Retsch GmbH, Haan, Germany) to pass through a 1 mm mesh screen and stored at −20 °C until analysis.

Moisture content was calculated from the weight loss during freeze-drying and expressed as a percentage of the original weight. Ash content was determined by heating 2 g of dried, powdered meat in a porcelain crucible at 600 °C for 8 h in a muffle furnace (Model L9/11, Nabertherm GmbH, Lilienthal, Germany); results are expressed as a percentage of the original weight.

Crude protein was analyzed by the macro-Kjeldahl method [46] using a digestion and distillation unit (Kjeltec 8400, Foss Analytical, Hillerød, Denmark). Approximately 1 g of dry meat sample was digested with concentrated sulfuric acid and a copper catalyst, then distilled and titrated; protein content is expressed as a percentage of the dry matter.

Total lipids were extracted with petroleum ether in a Soxhlet apparatus (Soxtherm 414, Gerhardt GmbH & Co. KG, Königswinter, Germany) for approximately 8 h. After solvent evaporation, fat content is expressed as a percentage of the dry matter.

Collagen content was determined by measuring hydroxyproline after acid hydrolysis, following the method of Woessner [48]. Briefly, approximately 0.5 g of freeze-dried meat was hydrolyzed in 6 M HCl at 110 °C for 24 h. The hydrolysate was neutralized, and hydroxyproline was quantified colorimetrically using a spectrophotometer (Model UV-1800, Shimadzu Corporation, Kyoto, Japan). Collagen content was calculated using a hydroxyproline-to-collagen conversion factor of 7.25 and expressed as a percentage of fresh meat weight.

### 2.13. Carcass Shrinkage Losses

In order to evaluate the shrinkage losses that occur during the chilling process, the weight of the carcasses was recorded immediately after slaughter (hot carcass weight) and after 24 h of chilling at 4 °C (cold carcass weight). Shrinkage losses were calculated by determining the difference between the hot and cold carcass weights expressed as a percentage.

### 2.14. Statistical Analysis

All statistical analyses were performed using SAS (V. 9.4). Data were tested for normality utilizing the Shapiro–Wilk-test and for homogeneity of variance using Levene’s test prior to analysis. Then, we examined the traits measured in both sexes with a two-way ANOVA. The model included breed, sex and their interaction as fixed effects:

(1) Y_ijk_ = μ + B_i_ + S_j_ + (B × S)_ij_ + E_ijk_

where Y_ijk_: is the observed trait, μ is the overall mean, B_i_ is the fixed effect of breed (i), S_j_ is the fixed effect of sex (j), (B × S)_ij_ is the interaction between breed and sex, and E_ijk_ is the random error term.

The reported values are means ± SD, and the *p*-values shown are derived from the ANOVA models. For reproductive organ measurements, which are sex-specific, we ran one-way ANOVAs within each sex to assess breed effects. Then we followed up with Tukey’s Honestly Significant Difference (HSD) test for post hoc pairwise comparisons for all variables where significant main effects (breed, sex, or their interaction) were detected. Statistical significance was set at *p* < 0.05. To control the expected proportion of false positives among the many comparisons, we corrected *p*-values for related traits using the Benjamini–Hochberg false discovery rate (FDR) method. Exact *p*-values are reported in the tables; values below 0.001 are denoted as *p* < 0.001.

## 3. Results

### 3.1. Carcass Traits and Primal Cut Weights

Significantly, breed affiliation was found to affect most of the carcass traits, with unique phenotypic features observed across the three genetic groups (Table 1 and Table 2). In the case of cold carcass weight, it was observed that the crossbred BAR × RAH group exhibited the highest overall mean value (30.12 ± 2.43 kg), which was significantly higher than the values observed in the purebred RAH and BAR groups (29.08 ± 2.71 kg and 22.79 ± 2.40 kg), respectively. This trend, where the crossbred exhibited superiority in weight-related traits, was consistently observed across the group in the case of final live body weight and slaughter weight, as the crossbred group exhibited the highest overall mean values for these traits (59.46 ± 1.28 kg and 58.27 ± 2.19 kg, respectively), with RAH group falling somewhere in between and BAR group registering the least values for these traits.

A notable exception to this general trend was, however, observed in the hot carcass weight including the tail, wherein RAH breed was found to possess a significantly higher overall mean value, a fact largely influenced by the extremely high values obtained for male animals, likely because of the very high fat tail trait of this particular breed.

On the other hand, an analysis of the primal cut weights from the left carcass side (Table 2) indicated that crossbred animals generally maintained their position of superiority by showing the overall highest weight values for the round cut (4.00 ± 0.77 kg), shoulder cut (2.79 ± 0.45 kg), and neck cut (2.61 ± 0.19 kg). However, RAH animals showed the overall highest weight value for the loin cut (2.66 ± 0.58 kg), while for the thoracic cut, overall weight values for BAR and crossbred animals were comparable and significantly higher than those of RAH animals.

### 3.2. Organ Weights

Breed identity had a significant impact on internal organ weights (Table 3). After data verification, RAH males and females showed similar organ weights, consistent with patterns observed in BAR and crossbred animals. For example, RAH male heart weight was 189.63 ± 4.33 g, while RAH female was 186.50 ± 4.20 g. RAH male liver weight was 890.08 ± 3.37 g, while RAH female was 888.99 ± 2.78 g. RAH male kidney weight was 170.35 ± 5.22 g, while RAH female was 171.06 ± 3.16 g. RAH male lung with trachea weight was 560.77 ± 8.92 g, while RAH female was 562.74 ± 12.23 g. RAH male spleen weight was 89.17 ± 2.88 g, while RAH female was 89.43 ± 2.76 g. On the other hand, in BAR and crossbred animals, consistent weights for each sex were recorded with minimum variation in each breed.

For liver weight, the crossbred animals recorded the highest weight, significantly larger than the weights recorded in the other animals, at 985.05 ± 2.50 g. BAR animals recorded the least weight in each of the internal organ weight categories.

### 3.3. Reproductive Organ Measurements

Analysis of the dimensions and weights of the reproductive tracts showed distinct breed differences (Table 3). For the males, testicular weight was greatly varied between breeds, with the crossbred animals bearing the highest testicular weights (455.31 ± 3.08 g), followed by RAH (350.00 ± 8.50 g) and BAR (327.97 ± 5.93 g).

For the females, the crossbreds showed the highest values for all the female reproductive tract weights. The uterine weight was the highest in the crossbreds, followed by ovarian weight, and the ovarian tubes, although these were comparable to those of RAH females. For the linear dimensions of the female reproductive organs, the crossbreds recorded the longest ovarian tubes and uterine horns, although these were comparable to those of RAH females. However, BAR females recorded the least weight for all the female reproductive organs, with the ovarian tubes and uterine horns being the least, such as 18.36 ± 0.27 cm and 13.09 ± 0.26 cm, respectively.

### 3.4. Fat Deposition Patterns

The adiposity parameters were found to vary depending on the breed; thus, it was observed that RAH sheep had the highest fat deposition. The highest amount of total fat was observed in RAH sheep at 2.72 ± 0.27 kg, while the highest amount of fat in the tail was observed at 2.59 ± 0.12 kg. The highest amount of gut fat was observed at 503.30 ± 12.96 g. In all fat reserves, RAH sheep had the highest fat deposition compared with the other two genetic groups. On the contrary, the fat reserves of the crossbred and BAR sheep were found to be lower and statistically similar.

The weight of the dissected fat from the components of the carcass was found to be much higher in RAH sheep (5.31 ± 0.23 kg) compared to BAR (3.56 ± 0.29 kg) and crossbred (3.90 ± 0.18 kg) sheep. Therefore, RAH sheep had the highest percentage of dissected fat and, on the other hand, had the lowest lean-to-fat ratio among the other three genetic groups. This indicates that RAH sheep had the highest fat deposition tendency in the fat component of the carcass. This may be related to the adaptive physiology and breed history of RAH sheep.

On the other hand, the crossbred stock had the highest REA, which is the cross-sectional area of the *Longissimus dorsi* muscle. This had an overall mean value of 16.79 ± 0.38 cm^2^, which was comparable to that of RAH sheep but was higher than that of BAR (14.68 ± 0.48 cm^2^), indicating that the muscular development was better in crossbred stock despite the lower fat content (Table 4).

### 3.5. Carcass Composition and Tissue Distribution

In relation to the overall carcass composition (Table 5), the crossbred animals had the highest trimmed meat weight (23.70 ± 1.35 kg) and trimmed meat percentage (74.06 ± 2.58%), which is an indication of the efficiency of meat yield. This implies that the crossbreeding of BAR with RAH has the potential for improved lean tissue mass compared to overall carcass weight. The bone weight was the heaviest among the crossbred animals overall (4.33 ± 0.26 kg). However, the differences among the breeds diminished when the bone weight was expressed as a percentage of the carcass weight.

The lean-to-fat ratio, which is an important measure of carcass quality, was the highest in BAR animals (4.12 ± 0.92). This was followed by the crossbred (3.38 ± 0.73) and the lowest in RAH (2.34 ± 0.44). This order is an indication of the overall effect of breed on the lean tissue mass and fat content. BAR animals had the best lean tissue mass compared to the overall carcass weight.

### 3.6. Gastrointestinal Tract (GIT) Characteristics and Blood Volume

Breed had a significant influence on GIT measurements (Table 6). The weights of the full GIT were significantly higher in crossbred and RAH animals than those of BAR animals. These differences might be attributed to differences in gut fill and tissue weights. On the other hand, the weights of the empty intestines were statistically similar for crossbred and RAH animals, which were significantly higher than those of BAR animals. These differences might be attributed to differences in intestinal tissue weights. The intestinal length was also significantly higher in crossbred animals (41.17 ± 2.18 m), followed by RAH (38.02 ± 1.82 m), and then BAR (34.87 ± 1.06 m).

The weights of the head, kidney, and glands (HKG) complex, which comprise the head, kidneys, and glands, were significantly higher in RAH animals (840.77 ± 15.82 g) than those of crossbred and BAR animals, which were statistically similar. These differences might be attributed to differences in head and gland weights.

The blood volume measurements showed that crossbred and RAH animals had significantly higher blood volume (4.45 L) than BAR animals (3.51 ± 0.24 L). These differences might be attributed to differences in animal sizes, with crossbred and RAH animals being larger than BAR animals.

### 3.7. Meat Chemical Composition

The proximate composition of the meats from the *Longissimus dorsi* muscle of the three breeds of sheep was significantly different (Table 7). Crossbred sheep had the highest percentage of moisture (74.66 ± 1.99%) and crude protein (21.65 ± 0.57%) contents in the meat. On the other hand, BAR and RAH meats had a significantly higher percentage of total lipid (fat) content (3.44% and 3.75%, respectively) than the crossbred meat (2.81 ± 0.39%). It is physiologically expected that fat would replace moisture.

The percentage of ash content, which refers to the content of minerals in the meat, was considerably higher in BAR meat (1.00 ± 0.05%) compared to the other two types of meat, which were almost comparable and statistically similar to each other.

The content of collagen was considerably lower in the crossbred meat (2.13 ± 0.34%), compared to the other two types of purebred meat. This may be due to a difference in the tenderness of the meat of the three types of sheep, as the tenderness of cooked meat is inversely proportional to the content of collagen.

### 3.8. Gastrointestinal Tract pH

From these measurements, breed-wise variation in pH was observed along the GIT (Table 8). For the ruminal pH, crossbred sheep showed the highest pH (6.87 ± 0.05), followed by RAH (6.85 ± 0.05), whereas BAR showed the lowest pH (6.47 ± 0.06) in the rumen environment.

For jejunal pH, crossbred sheep showed the lowest pH (6.34 ± 0.06), whereas RAH showed the highest jejunal pH (6.62 ± 0.06), with BAR showing intermediate values. For caecal pH, RAH showed the highest pH (6.81 ± 0.06), whereas BAR showed the lowest pH (6.39 ± 0.06), with crossbred sheep showing intermediate values (6.71 ± 0.06) in the caecal region. These pH variations in different regions of the GIT may vary across breeds and could be responsible for differences in growth performance and carcass characteristics.

### 3.9. Breed Effects

Collectively, these results demonstrate that breed significantly influences a comprehensive range of phenotypic traits encompassing carcass dimensions, tissue distribution, organ weights, reproductive characteristics, fat deposition patterns, meat composition, and gastrointestinal physiology. BAR × RAH animals exhibited superior performance in many weight-related and meat yield traits, while RAH animals demonstrated enhanced fat deposition and marked sexual dimorphism in organ weights. BAR animals, though smaller in total size, had favorable lean-to-fat ratios as well as meat compositional characteristics. The results offer a complete characterization that could be useful for planning breeding as well as production involving these resources.

## 4. Discussion

Crossbreeding is an effective strategy to improve growth, carcass, meat, and reproductive traits by exploiting heterosis, as demonstrated in various sheep breeds [16,17,18,19]. The current study presents a comprehensive phenotypic description of carcass characteristics, organ weights, reproductive organ dimensions, tissue chemical composition, and gastrointestinal characteristics of BAR, RAH, and BAR × RAH sheep. These detailed measurements directly addressed the study objective of characterizing breed and crossbreeding effects to support informed breeding decisions. The main results revealed that crossbred animals exhibited superior growth and meat yield traits, RAH purebreds displayed the highest fat deposition capacity, and BAR animals maintained the most favorable lean-to-fat ratio and distinct meat composition.

### 4.1. Carcass Traits and Primal Cut Weights

The fact that crossbred BAR × RAH recorded the highest cold carcass weight, final live body weight and slaughter weight compared to the two purebred groups supports the universally acknowledged fact that heterosis exists in crossbreeding schemes. Qiao et al. [16] studied the comparison of body conformation and carcass characteristics of Tibetan sheep and their crossbred progeny. Their study proved the fact that crossbred lambs possess significantly better body conformation and carcass characteristics, including live weight and carcass weight. They concluded that the heterosis of crossbred progeny resulted from the complementary combination of the genetic potential of the parental breeds. In another study, Wang et al. [19] proved the fact that crossbreeding between White-headed Suffolk sheep and Small-tailed Han sheep for two generations recorded a remarkable improvement in growth performance and slaughter characteristics.

Rabaa et al. [5] recently established significant breed effects on growth performance in Egyptian sheep reared under intensive production systems, showing that while BAR lambs had similar monthly live body weights to RAH lambs during certain growth periods, they had lower final body weights. They attributed this to the evolutionary adaptation of BAR sheep to survive and reproduce in harsh desert environments, which would not necessarily prioritize growth potential but rather survival and reproduction under nutritional stress conditions. Aboul-Naga et al. [4] further stated that the genomic structure of BAR sheep contains genes related to heat tolerance and nutritional stress adaptation that might influence the growth performance of this breed relative to RAH breed.

The notable exception observed for hot carcass weight including the tail, wherein RAH animals displayed a significantly greater overall mean with pronounced sexual dimorphism, reflects the characteristic fat tail morphology of this breed. In a research carried out by Yıldırım et al. [27] on the characteristics of the GIT of six breeds of Turkish sheep, including fat-tailed breeds like Akkaraman, Morkaraman and Awassi, the results showed that the fat-tailed breeds had significantly different carcass composition patterns compared with the thin-tailed breeds. The fat tail is an important energy reserve contributing not only to carcass weight but also to body condition, mainly in males.

When examining primal cut weights, the superior performance of crossbred animals for the round, shoulder and neck cuts indicates enhanced muscular development in these anatomical regions. This finding is supported by the findings of Qiao et al. [16] who stated that crossbred lambs had larger loin eye areas and better muscle development than purebred Tibetan sheep. The increased loin weight of RAH animals might be attributed to the unique muscle growth patterns of the breed, while the comparable thoracic region weight of BAR and crossbred animals might indicate that crossbreeding with RAH does not necessarily improve all muscle masses.

### 4.2. Organ Weights

Breed had a significant impact on internal organ weights (Table 3). This is in agreement with the study of Yıldırım et al. [27], who found significant differences in organ weights among Turkish sheep breeds, where Akkaraman lambs had heavier gastrointestinal organ weights compared with Awassi, Karayaka and Morkaraman lambs.

The fact that the crossbred animals had the highest mean for liver weight compared to the purebred animals could be an indication of heterotic effect on the development of metabolic organs. The liver is an important organ in the metabolism of nutrients, protein synthesis, as well as the regulation of energy. The increase in liver weight could, therefore, indicate an increase in the potential for metabolism. According to Qiao et al. [16], the crossbred lambs had improved feed efficiency and growth rates. This could have been an indication of the improved potential for metabolism. The lowest overall mean for liver weight was found in BAR animals. This is an indication of the overall smaller body size of BAR animals. This could have been an evolutionary adaptation for survival in an environment with limited energy availability.

### 4.3. Reproductive Organ Measurements

The evident breed-specific characteristics of reproductive tract dimensions and weights have significant implications for breed fertility and reproductive management. A comparative study on the biometry of the testes in three breeds of Nigerian sheep (Uda, Balami and Yankasa) was carried out by Ibrahim et al. [49]. The results of the study showed significant breed differences in scrotal circumference, weight of the testes, volume of the testes, and weight of the epididymis. The Uda breed had significantly higher values for the parameters studied. The biometrical characteristics of the testes are indicative of high spermatozoa production per unit mass of the testes and epididymis, which is associated with high fertility.

Among the female category, the consistent superiority of the crossbred animals for all the reproductive tract measurements, could be attributed to better development of the reproductive organs, which might result in better reproductive efficiency. Kamalzadeh et al. [30] carried out intensive studies on the variations in the patterns of follicle development in prolific and non-prolific breeds of sheep. They reported that Romanov ewes achieved high ovulation rates through a more numerous population of recruitable follicles with a similar incidence of selection through atresia, while Finn ewes achieved high ovulatory potential through a markedly reduced incidence of selection through atresia. The findings align with those of Ibrahim et al. [49], who reported significant breed differences in reproductive organ morphometry and suggested that such biometrical characteristics can be used by farmers in selecting good breeding animals for genetic improvement.

### 4.4. Fat Deposition Patterns

The marked breed differences in fat deposition patterns indicate basic differences in breed physiology. The GIT characteristics of fat-tailed and thin-tailed Turkish sheep breeds have been investigated by Yıldırım et al. [27]. Fat-tailed sheep breeds, including Morkaraman, Akkaraman and Awassi sheep breeds, have shown different fat deposition characteristics compared with thin-tailed sheep breeds. Fat-tailed sheep have evolved with the ability to conserve energy in the tail region of the body in response to seasonal feed supply and environmental stress. This evolutionary adaptation may explain the enhanced fat deposition observed in RAH animals in the present study, as the breed originated and developed in the Nile Delta region where seasonal variations in feed quality and quantity would have favored individuals capable of storing energy during periods of abundance for utilization during scarcity.

Such a trend is desirable for the quality of the carcass, as excessive fat content is undesirable. Wang et al. [19] found that crossbred lambs from White-headed Suffolk and Small-tailed Han sheep breeds showed better meat quality characteristics. Such a trend indicates that crossbreeding can be used to optimize fat deposition rather than increasing fat content.

The inverse association between fat deposition and lean-to-fat ratio suggests that nutrient partitioning priorities differ by breed. Alkass et al. [26] reviewed allometric growth coefficients of carcass and non-carcass components in small ruminants and reported that bone tissue is precocious (b < 1), demonstrating a declining proportion with increasing carcass and empty body weight, while lean tissue also exhibits growth coefficients below unity. Subcutaneous fat grows at a higher rate than intramuscular fat, while non-carcass fat depots mature early.

### 4.5. Gastrointestinal Tract (GIT) Characteristics and Blood Volume

Significant breed effects on measurements of the GIT, including full and empty GIT weights, intestinal length, and tissue mass, have significant implications for digestive efficiency and nutrient utilization. A comprehensive study on the characteristics of the GIT of six Turkish sheep breeds by Yıldırım et al. [27] found that Akkaraman lambs had heavier GIT weights than Awassi, Karayaka, and Morkaraman breeds.

### 4.6. Meat Chemical Composition

Significant differences in the chemical composition of meat between different breeds have great implications for nutritional value, sensory qualities, and acceptability of meat. The present study found that crossbred animal meat possessed the highest moisture content, and the meat of BAR and RAH possessed more total lipids compared to crossbred animal meat. The present findings correlate with the inverse relationship between moisture and total lipid content in meat.

The lower collagen content found in crossbred meat compared to purebred meat could indicate differences in meat tenderness, as collagen content is inversely proportional to tenderness in cooked meat products.

### 4.7. Gastrointestinal Tract (GIT) pH

The unique breed-specific variations in pH values of GIT offer new insights into the differences in the digestive physiology of breeds, indicates a difference in the fermentation patterns, volatile fatty acids, or salivary buffers of the breeds. Yıldırım et al. [27] found that the pH value of the rumen was higher in Turkish Merino and Akkaraman lambs than in Karayaka and Morkaraman lambs, indicates that there are indeed differences in the pH value of the rumen among breeds, even under similar feeding conditions. The pH value in the rumen, according to Yıldırım et al. [27], affects the microbes, fermentation patterns, and efficiency of utilization of feedstuffs.

The regional variations found for jejunal and caecal pH, indicate that the digestive physiology of GIT varies with the breed. Significant breed differences for intestinal pH and digestive enzyme activities were found by Ding et al. [40] for the Small-tailed Han sheep and the Tan sheep, with the pH value for the duodenum, jejunum, and ileum sections showing significant variations between the breeds. According to the authors, the breed-specific patterns for the gastrointestinal pH value can account for the variations found for the digestive enzyme activities, nutrient absorption, and the composition of the intestinal microflora. According to the study conducted by Ranilla et al. [37], breed differences for the ruminal activity are found even under identical dietary conditions, with the highest in situ degradability of forages found for Churra sheep compared with the Merino sheep.

### 4.8. Implications for Breeding and Production

The comprehensive phenotypic characterization achieved by this current study has significant implications for the breeding programs and production systems of sheep in Egypt. The outstanding performance of crossbred animals between the two breeds, BAR × RAH, for all weight-related traits and meat yield parameters indicates that crossbreeding between the two breeds has the potential to produce crossbred animals that possess the excellent adaptation and hardiness of BAR breed while simultaneously inheriting the excellent growth potential of RAH breed. This observation is consistent with the findings of Wang et al. [19], who demonstrated that crossbreeding programs can be developed to achieve the objectives of simultaneously improving growth performance and ecological sustainability.

The significant breed differences for fat deposition patterns, with RAH animals showing the highest fat content and BAR animals showing the most favorable lean-to-fat ratios, indicate that market demands and consumer preferences should be taken into consideration during selection and breeding programs for the two breeds. Aboul-Naga et al. [4] pointed out that the high within-breed genetic variation in BAR sheep offers opportunities for selection to improve growth rates of lambs while simultaneously maintaining moderate ewe size, lean meat content, and heat resilience.

Significant variation between the breeds for reproductive organ measurements indicates that there may be variations in reproductive capacity that may affect the fertility and productivity of the flock. Ibrahim et al. [49] mentioned that testicular biometry can be used by farmers to select good breeding animals for genetic improvement programs, since testicular size is correlated with the capacity of sperm production and fertility.

Beyond digestive physiology, the economic and agricultural relevance of the breed differences observed in this study warrants consideration. BAR and RAH were selected for their complementary traits: BAR is adapted to arid environments and produces lean meat under marginal conditions [4,5], while RAH grows faster and yields higher carcass weights in intensive systems [10,13]. Their crossbred combines these attributes, offering a balance between productivity and adaptability. Nutritionally, BAR and crossbred meat contain less fat and more ash, supporting leaner, more nutrient-dense products. For Egyptian agriculture, these findings support a strategy of using BAR on marginal lands, RAH in productive areas, and crossbreeding to meet mutton demand while conserving indigenous breeds [50,51]. This integrated approach can enhance food security, improve livelihoods of smallholder farmers, and reduce the environmental footprint of sheep production by better matching breed capabilities to production environments.

Finally, the findings of this study are based on animals of a similar age, raised under a single management system at one geographic location, which may limit their generalizability to other ages, production environments, or climatic conditions. While the phenotypic differences observed were consistent and robust, validation across broader populations would strengthen their applicability. Future work employing molecular approaches could further clarify the mechanisms underlying these breed differences.

## 5. Conclusions

This study provides a comprehensive baseline of phenotypic variation in carcass, organ, reproductive, meat quality, and gastrointestinal traits among Barki (BAR), Rahmani (RAH), and their crossbred (BAR × RAH) sheep. The crossbred animals consistently outperformed the purebreds in weight-related traits, including cold carcass weight (30.12 kg), final live weight (59.46 kg), and trimmed meat weight (23.70 kg), while also exhibiting the heaviest testes (455.31 g), uterus (20.49 g), and longest intestines (41.17 m). Purebred RAH animals displayed the greatest fat deposition, with the highest total fat (2.72 kg) and fat tail weight (2.59 kg). In contrast, BAR animals had the leanest carcasses (lean-to-fat ratio 4.12) and the highest meat ash content (1.00%). These distinct phenotypic profiles provide a robust basis for genetic improvement programs. Future research should validate these phenotypic findings in larger and more diverse populations and incorporate molecular approaches to uncover the genetic and physiological mechanisms underlying the observed breed differences.

## Figures and Tables

**Table 1:** Comprehensive summary of morphological carcass traits for Barki (BAR), Rahmani (RAH) and BAR × RAH sheep breeds.

Trait	Unit	Sex	BAR	RAH	BAR × RAH	*p*-Value
Cold carcass weight	kg	M	22.13 ± 3.01 ^c^	29.29 ± 2.55 ^b^	30.67 ± 2.25 ^a^	<0.001
		F	23.44 ± 1.67 ^b^	28.88 ± 3.14 ^a^	29.56 ± 2.72 ^a^	0.005
		Overall	22.79 ± 2.40 ^c^	29.08 ± 2.71 ^b^	30.12 ± 2.43 ^a^	<0.001
Final live body weight	kg	M	47.41 ± 2.95 ^c^	58.96 ± 1.43 ^b^	60.44 ± 1.36 ^a^	0.009
		F	46.17 ± 1.86 ^b^	58.02 ± 1.37 ^a^	58.49 ± 1.18 ^a^	0.002
		Overall	46.79 ± 1.70 ^c^	58.49 ± 1.34 ^b^	59.46 ± 1.28 ^a^	<0.001
Slaughter weight	kg	M	46.29 ± 2.81 ^b^	58.96 ± 2.50 ^a^	59.34 ± 2.22 ^a^	<0.001
		F	44.91 ± 2.93 ^c^	56.70 ± 2.43 ^a^	57.20 ± 2.16 ^b^	0.001
		Overall	45.60 ± 2.75 ^c^	57.38 ± 2.44 ^b^	58.27 ± 2.19 ^a^	<0.001
Empty body weight	kg	M	33.16 ± 2.77 ^b^	40.75 ± 1.04 ^c^	40.76 ± 2.50 ^a^	<0.001
		F	33.04 ± 2.40 ^c^	40.75 ± 2.35 ^a^	40.53 ± 1.21 ^b^	<0.001
		Overall	33.10 ± 2.49 ^c^	40.75 ± 5.26 ^b^	40.64 ± 1.87 ^a^	<0.001
Hot carcass weight	kg	M	24.40 ± 1.25 ^c^	30.97 ± 0.74 ^b^	31.61 ± 0.23 ^a^	<0.001
		F	24.41 ± 0.90 ^c^	33.68 ± 0.86 ^b^	32.55 ± 0.80 ^a^	<0.001
		Overall	24.41 ± 1.05 ^c^	32.33 ± 1.50 ^b^	32.08 ± 0.75 ^a^	<0.001
Hot carcass weight—tail	kg	M	23.07± 0.71 ^c^	28.35 ± 0.74 ^b^	30.02 ± 0.48 ^b^	<0.001
		F	23.18 ± 1.00 ^c^	31.11 ± 0.86 ^a^	30.79 ± 0.84 ^b^	<0.001
		Overall	23.12± 1.07 ^c^	29.73 ± 1.50 ^a^	30.41 ± 0.85 ^b^	<0.001
Dressing “1” ^1^	%	M	53.06 ± 2.54 ^b^	48.12 ± 2.86 ^a^	54.23 ± 2.97 ^b^	<0.001
		F	55.48 ± 2.90 ^a^	55.50 ± 2.40 ^a^	57.02 ± 2.78 ^a^	0.854
		Overall	54.27 ± 2.14 ^b^	51.81± 10.38 ^a^	55.62 ± 2.40 ^b^	0.002
Dressing “2” ^2^	%	M	73.78 ± 2.15 ^b^	71.90± 1.16 ^c^	77.79 ± 4.75 ^a^	<0.001
		F	77.29 ± 3.32 ^b^	76.48 ± 2.83 ^b^	80.35 ± 1.68 ^a^	0.035
		Overall	75.53 ± 4.13 ^b^	74.19 ± 30.67 ^c^	79.07 ± 3.61 ^a^	<0.001
Carcass width at third rib	cm	M	16.61 ± 1.08 ^c^	23.78 ± 0.51 ^a^	19.77 ± 0.54 ^b^	<0.001
		F	16.59 ± 1.24 ^c^	17.80 ± 1.03 ^b^	18.72 ± 1.23 ^a^	0.019
		Overall	16.60 ± 1.12 ^c^	20.79 ± 3.14 ^a^	19.24 ± 1.14 ^b^	<0.001
Carcass width at seventh rib	cm	M	21.82 ± 1.06 ^c^	24.46 ± 1.66 ^a^	23.28 ± 1.09 ^b^	0.01
		F	21.51 ± 0.82 ^c^	23.72 ± 0.65 ^a^	23.59 ± 0.66 ^b^	<0.001
		Overall	21.66 ± 0.93 ^c^	24.09 ± 1.27 ^a^	23.44 ± 0.88 ^b^	<0.001
Round length of carcass	cm	M	22.41 ± 0.79 ^b^	24.19 ± 1.27 ^a^	24.80 ± 1.25 ^a^	<0.001
		F	22.49 ± 1.13 ^c^	25.02 ± 1.43 ^b^	25.16 ± 1.35 ^a^	0.004
		Overall	22.45 ± 0.94 ^c^	24.60 ± 1.30 ^a^	24.98 ± 1.27 ^b^	<0.001
Round circumference of carcass	cm	M	43.74 ± 1.79 ^b^	46.64 ± 1.78 ^a^	47.49 ± 1.55 ^a^	<0.001
		F	43.42 ± 1.46 ^c^	46.00 ± 1.59 ^b^	47.66 ± 2.33 ^a^	0.003
		Overall	43.58 ± 1.58 ^b^	46.32 ± 1.70 ^a^	47.58 ± 1.92 ^a^	<0.001
Loin length of carcass	cm	M	20.95 ± 1.26 ^c^	23.35 ± 1.32 ^a^	24.92 ± 1.30 ^b^	<0.001
		F	21.93 ± 0.86 ^c^	22.83 ± 1.31 ^b^	24.99 ± 1.28 ^a^	<0.001
		Overall	21.44 ± 1.22 ^c^	23.59 ± 1.22 ^a^	24.96 ± 1.25 ^b^	<0.001
Carcass length	cm	M	52.61 ± 1.44 ^b^	55.64 ± 1.24 ^c^	57.96 ± 1.37 ^a^	<0.001
		F	53.29 ± 1.58 ^c^	55.52 ± 1.32 ^b^	58.60 ± 0.72 ^a^	<0.001
		Overall	52.95 ± 1.50 ^b^	55.58 ± 1.28 ^c^	58.28 ± 1.09 ^a^	<0.001

All animals were approximately 36.5 ± 0.75 months old at the start of the study, with no significant age difference among breeds or sexes. Values are presented as mean ± standard deviation (SD). ^a–c^ indicate significant differences among breeds within the same row. Means sharing the same letter are not significantly different (*p* > 0.05). ^1^ Dressing “1” = (Hot carcass weight/Slaughter weight) × 100. ^2^ Dressing “2” = (Hot carcass weight/Empty body weight) × 100.

**Table 2:** Carcass measurements and primal cuts weights for Barki (BAR), Rahman (RAH) and BAR × RAH sheep breeds.

Trait	Unit	Sex	BAR	RAH	BAR × RAH	*p*-Value
Cold carcass weight (left side, no tail)	kg	M	11.56 ± 0.49 ^b^	13.66 ± 0.87 ^c^	16.53 ± 0.29 ^a^	<0.001
		F	12.22 ± 0.96 ^b^	15.44 ± 0.68 ^a^	15.78 ± 0.45 ^a^	0.003
		Overall	11.89 ± 0.84 ^c^	14.55 ± 0.78 ^b^	16.16 ± 0.36 ^a^	<0.001
Round weight	kg	M	3.44 ± 0.49 ^b^	2.14 ± 0.37 ^c^	4.14 ± 0.59 ^a^	<0.001
		F	3.61 ± 0.62 ^a^	3.31 ± 0.22 ^a^	3.85 ± 0.94 ^a^	0.421
		Overall	3.52 ± 0.54 ^b^	2.72 ± 0.67 ^c^	4.00 ± 0.77 ^a^	<0.001
Loin weight	kg	M	2.08 ± 0.36 ^b^	3.13 ± 0.44 ^a^	2.84 ± 0.31 ^a^	0.001
		F	1.77 ± 0.37 ^b^	2.19 ± 0.32 ^a^	2.21 ± 0.52 ^a^	0.042
		Overall	1.93 ± 0.39 ^c^	2.66 ± 0.58 ^a^	2.52 ± 0.52 ^b^	<0.001
Thoracic region weight	kg	M	3.58 ± 0.35 ^a^	2.62 ± 0.39 ^c^	3.34 ± 0.64 ^b^	0.008
		F	2.89 ± 0.56 ^a^	3.09 ± 0.51 ^a^	3.27 ± 0.54 ^a^	0.532
		Overall	3.24 ± 0.56 ^a^	2.85 ± 0.50 ^b^	3.31 ± 0.57 ^a^	0.047
Shoulder weight	kg	M	2.59 ± 0.32 ^a^	2.23 ± 0.32 ^b^	2.94 ± 0.34 ^a^	0.007
		F	2.48 ± 0.30 ^a^	2.70 ± 0.43 ^a^	2.64 ± 0.51 ^a^	0.675
		Overall	2.54 ± 0.30 ^b^	2.46 ± 0.42 ^b^	2.79 ± 0.45 ^a^	0.039
Neck weight	kg	M	1.40 ± 0.21 ^b^	0.86 ± 0.05 ^c^	2.67 ± 0.17 ^a^	<0.001
		F	1.38 ± 0.23 ^b^	2.22 ± 0.19 ^a^	2.54 ± 0.20 ^a^	<0.001
		Overall	1.39 ± 0.21 ^c^	1.54 ± 0.68 ^b^	2.61 ± 0.19 ^a^	<0.001
Flank weight	kg	M	0.63 ± 0.07 ^b^	0.80 ±0.08 ^a^	0.92 ± 0.07 ^a^	<0.001
		F	0.63 ± 0.06 ^b^	0.85 ± 0.07 ^a^	0.93 ± 0.07 ^a^	<0.001
		Overall	0.63 ± 0.06 ^b^	0.80± 00.06 ^a^	0.92 ± 0.07 ^a^	<0.001

Values are presented as mean ± standard deviation (SD). ^a–c^ indicate significant differences among breeds within the same row. Means sharing the same letter are not significantly different (*p* > 0.05).

**Table 3:** Organ weights and reproductive traits for Barki (BAR), Rahmani (RAH) and BAR × RAH sheep breeds.

Trait	Unit	Sex	BAR	RAH	BAR × RAH	*p*-Value
Heart weight	g	M	157.57 ± 2.64 ^b^	189.63 ± 4.33 ^a^	190.07 ± 3.86 ^a^	<0.001
		F	158.43 ± 2.84 ^b^	186.50 ± 4.20 ^a^	191.78 ± 3.46 ^a^	<0.001
		Overall	158.00 ± 2.69 ^c^	188.07 ± 3.12 ^b^	190.92 ± 4.10 ^a^	<0.001
Liver weight	g	M	788.50 ± 2.60 ^b^	890.08 ± 3.37 ^a^	984.66 ± 2.07 ^a^	<0.001
		F	785.90 ± 3.24 ^b^	888.99 ± 2.78 ^a^	985.43 ± 2.95 ^a^	<0.001
		Overall	787.20 ± 3.40 ^b^	889.54 ± 2.91 ^a^	985.05 ± 2.50 ^a^	<0.001
Kidney weight	g	M	111.90 ± 1.38 ^b^	170.35 ± 5.22 ^a^	172.78 ± 2.14 ^a^	<0.001
		F	108.25 ± 3.58 ^c^	171.06 ± 3.16 ^b^	174.07 ± 1.98 ^a^	<0.001
		Overall	110.08 ± 3.24 ^c^	170.71 ± 3.84 ^b^	173.43 ± 2.34 ^a^	<0.001
Lung + trachea weight	g	M	489.44 ± 9.26 ^b^	560.77 ± 8.92 ^a^	580.93 ± 8.46 ^a^	<0.001
		F	502.80 ± 11.85 ^b^	562.74 ± 12.23 ^a^	576.77 ± 9.94 ^a^	<0.001
		Overall	496.12 ± 11.77 ^b^	561.76 ± 10.58 ^a^	578.85 ± 9.22 ^a^	<0.001
Spleen weight	g	M	78.70 ± 1.57 ^b^	89.17 ± 2.88 ^a^	93.59 ± 2.38 ^a^	<0.001
		F	78.20 ± 1.52 ^c^	89.43 ± 2.76 ^b^	93.16 ± 2.02 ^a^	<0.001
		Overall	78.45 ± 1.50 ^c^	89.30 ± 2.64 ^b^	93.38 ± 2.24 ^a^	<0.001
Testis weight	g	M	327.97 ± 5.93 ^b^	350.00 ± 8.50 ^b^	455.31 ± 3.08 ^a^	<0.001
Female Reproductive System		F	37.99 ± 0.34 ^c^	39.68 ± 0.52 ^b^	40.04 ± 0.12 ^a^	<0.001
Weight of ovaries	g	F	1.45 ± 0.12 ^b^	1.68 ± 0.15 ^a^	1.72 ± 0.14 ^a^	0.038
Weight of ovarian tubes	g	F	1.79 ± 0.03 ^c^	1.94 ± 0.04 ^b^	2.08 ± 0.08 ^a^	<0.001
Uterine weight	g	F	19.18 ± 0.37 ^c^	20.31 ± 0.34 ^b^	20.49 ± 0.16 ^a^	<0.001
Length of ovarian tubes	cm	F	18.36 ± 0.27 ^b^	19.79 ± 0.55 ^a^	19.64 ± 0.58 ^a^	<0.001
Length of uterine horns	cm	F	13.09 ± 0.26 ^b^	14.52 ± 0.54 ^a^	14.37 ± 0.53 ^a^	<0.001

Values are presented as mean ± standard deviation (SD). ^a–c^ indicate significant differences among breeds within the same row. Means sharing the same letter are not significantly different (*p* > 0.05).

**Table 4:** Fat measurements and rib eye area for Barki (BAR), Rahmani (RAH) and BAR × RAH sheep.

Trait	Unit	Sex	BAR	RAH	BAR × RAH	*p*-Value
Total fat stores	kg	M	2.23 ± 0.32 ^b^	2.67 ± 0.27 ^a^	2.35 ± 0.17 ^b^	0.048
		F	2.32 ± 0.33 ^a^	2.77 ± 0.28 ^a^	2.30 ± 0.15 ^a^	0.067
		Overall	2.28 ± 0.31 ^b^	2.72 ± 0.27 ^a^	2.33 ± 0.16 ^b^	0.002
Heart fat weight (pericardial fat)	g	M	89.25 ± 1.20 ^b^	101.74 ± 2.50 ^a^	92.56 ± 2.61 ^b^	<0.001
		F	85.80 ± 3.41 ^b^	99.35 ± 2.86 ^a^	89.62 ± 2.28 ^b^	<0.001
		Overall	87.52 ± 3.15 ^c^	100.55 ± 2.82 ^a^	91.09 ± 2.89 ^b^	<0.001
Kidney fat weight (pelvis and perinephric fat)	g	M	233.65 ± 4.94 ^a^	236.86 ± 8.88 ^a^	225.86 ± 5.63 ^b^	0.043
		F	228.86 ± 5.32 ^b^	237.06 ± 5.96 ^a^	227.09 ± 7.26 ^b^	0.024
		Overall	231.25 ± 5.49 ^ab^	236.96 ± 7.07 ^a^	226.48 ± 6.21 ^b^	0.005
Gut fat weight	g	M	479.69 ± 12.71 ^b^	502.39 ± 13.33 ^a^	481.86 ± 15.64 ^b^	0.041
		F	475.71 ± 8.46 ^b^	504.21 ± 13.96 ^a^	471.75 ± 15.25 ^b^	0.001
		Overall	477.70 ± 10.58 ^b^	503.30 ± 12.96 ^a^	476.81 ± 16.40 ^b^	<0.001
Fat tail weight	kg	M	1.33 ± 0.08 ^b^	2.62 ± 0.11 ^a^	1.59 ± 0.10 ^b^	<0.001
		F	1.23 ± 0.09 ^b^	2.57 ± 0.13 ^a^	1.76 ± 0.06 ^b^	<0.001
		Overall	1.28 ± 0.10 ^b^	2.59 ± 0.12 ^a^	1.57 ± 0.08 ^b^	<0.001
Gastrointestinal tract fat (omental and mesenteric fat)	g	M	897.47 ± 32.18 ^a^	941.28 ± 52.88 ^a^	770.76 ± 38.99 ^b^	<0.001
		F	890.44 ± 34.54 ^a^	943.12 ± 104.45 ^a^	790.37 ± 36.53 ^b^	0.003
		Overall	893.96 ± 32.05 ^a^	942.20 ± 77.78 ^a^	780.56 ± 38.76 ^b^	<0.001
Rib eye area (*Longissimus dorsi* area at 10th rib)	cm^2^	M	14.98 ± 0.38 ^b^	16.92 ± 0.41 ^a^	16.87 ± 0.40 ^a^	<0.001
		F	14.37 ± 0.35 ^b^	16.85 ± 0.43 ^a^	16.71 ± 0.32 ^a^	<0.001
		Overall	14.68 ± 0.48 ^b^	16.89 ± 0.41 ^a^	16.79 ± 0.38 ^a^	<0.001

Values are presented as mean ± standard deviation (SD). ^a–c^ indicate significant differences among breeds within the same row. Means sharing the same letter are not significantly different (*p* > 0.05).

**Table 5:** Physical assessment of carcass composition for Barki (BAR), Rahmani (RAH) and BAR × RAH sheep breeds.

Trait	Unit	Sex	BAR	RAH	BAR × RAH	*p*-Value
Bone weight	kg	M	3.49 ± 0.27 ^a^	4.11 ± 0.14 ^a^	4.64 ± 0.34 ^a^	0.051
		F	3.63 ± 0.37 ^a^	3.57 ± 0.19 ^a^	4.02 ± 0.27 ^a^	0.555
		Overall	3.56 ± 0.30 ^b^	3.84 ± 0.18 ^ab^	4.33 ± 0.26 ^a^	0.031
Bone percentage	%	M	14.32 ± 2.04 ^a^	13.29 ± 1.66 ^a^	14.65 ± 2.24 ^a^	0.646
		F	14.61 ± 3.15 ^a^	11.54 ± 2.82 ^a^	12.34 ± 2.23 ^a^	0.196
		Overall	14.46 ± 2.51 ^a^	12.41 ± 2.43 ^a^	13.49 ± 2.21 ^a^	0.165
Trimmed meat weight	kg	M	17.35 ± 1.05 ^b^	21.57 ± 1.34 ^a^	23.05 ± 1.15 ^a^	<0.001
		F	17.64 ± 1.08 ^b^	22.19 ± 1.64 ^a^	24.35 ± 1.29 ^a^	<0.001
		Overall	17.49 ± 1.04 ^c^	21.88 ± 1.47 ^b^	23.70 ± 1.35 ^a^	<0.001
Trimmed meat percentage	%	M	71.07 ± 1.10 ^a^	69.61 ± 1.35 ^a^	72.93 ± 2.63 ^a^	0.075
		F	71.10 ± 1.27 ^a^	71.27 ± 1.10 ^a^	75.19 ± 2.13 ^a^	0.064
		Overall	71.09 ± 1.21 ^b^	70.44 ± 1.26 ^b^	74.06 ± 2.58 ^a^	0.003
Dissected fat weight	kg	M	3.56 ± 0.28 ^b^	5.29 ± 0.19 ^a^	3.94 ± 0.18 ^b^	<0.001
		F	3.55 ± 0.33 ^b^	5.34 ± 0.27 ^a^	3.86 ± 0.17 ^b^	<0.001
		Overall	3.56 ± 0.29 ^b^	5.31 ± 0.23 ^a^	3.90 ± 0.18 ^b^	<0.001
Dissected fat percentage	%	M	14.61 ± 1.42 ^b^	17.10 ± 1.05 ^a^	12.46 ± 0.69 ^c^	<0.001
		F	14.29 ± 1.38 ^b^	17.19 ± 1.17 ^a^	11.94 ± 0.54 ^c^	<0.001
		Overall	14.45 ± 1.35 ^b^	17.14 ± 1.06 ^a^	12.20 ± 0.71 ^c^	<0.001
Lean: Fat ratio	-	M	4.08 ± 0.89 ^a^	2.51 ± 0.26 ^b^	3.55 ± 0.84 ^a^	0.007
		F	4.16 ± 1.02 ^a^	2.16 ± 0.52 ^b^	3.20 ± 0.59 ^a^	<0.001
		Overall	4.12 ± 0.92 ^a^	2.34 ± 0.44 ^c^	3.38 ± 0.73 ^b^	<0.001
Lean: Bone ratio	-	M	5.07 ± 0.87 ^a^	5.32 ± 1.01 ^a^	5.31 ± 1.40 ^a^	0.915
		F	5.12 ± 1.31 ^a^	6.52 ± 1.69 ^a^	5.93 ± 1.42 ^a^	0.298
		Overall	5.09 ± 1.06 ^b^	5.92 ± 1.47 ^a^	5.62 ± 1.38 ^ab^	0.231
Carcass weight to fasted weight	%	M	53.06 ± 1.54 ^a^	53.01 ± 1.14 ^a^	54.23 ± 1.97 ^a^	0.942
		F	55.48 ± 1.90 ^a^	55.30 ± 1.83 ^a^	57.07 ± 1.10 ^a^	0.826
		Overall	54.27 ± 1.14 ^a^	54.16 ± 1.79 ^a^	55.65 ± 1.52 ^a^	0.777
Net meat ratio	%	M	45.45 ± 1.77 ^a^	45.98 ± 1.37 ^a^	46.58 ± 1.54 ^a^	0.949
		F	47.39 ± 1.82 ^a^	48.92 ± 1.77 ^a^	47.60 ± 1.13 ^a^	0.879
		Overall	46.42 ± 1.71 ^a^	47.45 ± 1.50 ^a^	47.09 ± 1.16 ^a^	0.892

Values are presented as mean ± standard deviation (SD). ^a–c^ indicate significant differences among breeds within the same row. Means sharing the same letter are not significantly different (*p* > 0.05).

**Table 6:** Carcass shrink losses, gastrointestinal traits, and blood parameters for Barki (BAR), Rahmani (RAH) and BAR × RAH sheep breeds.

Trait	Unit	Sex	BAR	RAH	BAR × RAH	*p*-Value
FGIT	kg	M	5.77 ± 0.48 ^b^	8.30 ± 0.36 ^a^	8.51 ± 0.64 ^a^	<0.001
		F	5.20 ± 1.21 ^b^	7.83 ± 0.38 ^a^	8.12 ± 0.51 ^a^	<0.001
		Overall	5.49 ± 0.93 ^c^	8.07 ± 0.44 ^b^	8.32 ± 0.59 ^a^	<0.001
EGIT	kg	M	2.02 ± 0.18 ^b^	2.91 ± 0.13 ^a^	2.98 ± 0.20 ^a^	<0.001
		F	1.82 ± 0.40 ^b^	2.74 ± 0.13 ^a^	2.83 ± 0.16 ^a^	<0.001
		Overall	1.92 ± 0.31 ^c^	2.82 ± 0.15 ^b^	2.91 ± 0.19 ^a^	<0.001
Full stomach	kg	M	3.75 ± 0.28 ^b^	4.85 ± 0.37 ^a^	5.22 ± 0.42 ^a^	<0.001
		F	3.76 ± 0.22 ^b^	5.36 ± 0.43 ^a^	5.45 ± 0.46 ^a^	<0.001
		Overall	3.75 ± 0.24 ^c^	5.10 ± 0.47 ^b^	5.34 ± 0.43 ^a^	<0.001
Empty stomach	kg	M	1.16 ± 0.09 ^a^	0.97 ± 0.07 ^b^	1.22 ± 0.11 ^a^	<0.001
		F	1.05 ± 0.13 ^a^	1.07 ± 0.10 ^a^	1.30 ± 0.10 ^a^	0.003
		Overall	1.10 ± 0.12 ^b^	1.02 ± 0.10 ^b^	1.26 ± 0.11 ^a^	<0.001
Full intestine	kg	M	2.23 ± 0.13 ^b^	2.56 ± 0.18 ^a^	2.76 ± 0.17 ^a^	<0.001
		F	2.18 ± 0.23 ^b^	2.56 ± 0.23 ^a^	2.97 ± 0.20 ^a^	<0.001
		Overall	2.20 ± 0.18 ^c^	2.56 ± 0.19 ^b^	2.87 ± 0.21 ^a^	<0.001
Empty intestine	kg	M	0.91 ± 0.01 ^b^	1.12 ± 0.02 ^a^	1.14 ± 0.02 ^a^	<0.001
		F	0.93 ± 0.02 ^b^	1.13 ± 0.02 ^a^	1.14 ± 0.03 ^a^	<0.001
		Overall	0.92 ± 0.02 ^b^	1.12 ± 0.02 ^a^	1.14 ± 0.02 ^a^	<0.001
Intestine length	m	M	34.32 ± 1.04 ^c^	38.05 ± 2.42 ^b^	40.76 ± 1.85 ^a^	<0.001
		F	35.43 ± 0.80 ^c^	38.00 ± 1.20 ^b^	41.59 ± 2.53 ^a^	<0.001
		Overall	34.87 ± 1.06 ^c^	38.02 ± 1.82 ^b^	41.17 ± 2.18 ^a^	<0.001
HKG	g	M	802.62 ± 11.25 ^b^	841.00 ± 16.91 ^a^	800.34 ± 9.12 ^b^	<0.001
		F	790.37 ± 2.07 ^b^	840.53 ± 16.96 ^a^	791.90 ± 12.51 ^b^	<0.001
		Overall	796.49 ± 11.09 ^b^	840.77 ± 15.82 ^a^	796.12 ± 11.03 ^b^	<0.001
Blood volume	L	M	3.56 ± 0.29 ^b^	4.51 ± 0.52 ^a^	4.53 ± 0.54 ^a^	0.003
		F	3.46 ± 0.21 ^b^	4.35 ± 0.48 ^a^	4.39 ± 0.36 ^a^	<0.001
		Overall	3.51 ± 0.24 ^b^	4.43 ± 0.48 ^a^	4.46 ± 0.44 ^a^	<0.001
Carcass shrink losses	%	M	2.27 ± 3.77 ^a^	1.68 ± 2.88 ^a^	1.14 ± 2.67 ^a^	0.854
		F	1.37 ± 2.24 ^a^	2.23 ± 2.90 ^a^	2.19 ± 3.48 ^a^	0.879
		Overall	1.82 ± 3.01 ^a^	1.95 ± 2.78 ^a^	1.67 ± 3.01 ^a^	0.975

Values are presented as mean ± standard deviation (SD). ^a–c^ indicate significant differences among breeds within the same row. Means sharing the same letter are not significantly different (*p* > 0.05). FGIT: Full gastrointestinal tract weight. EGIT: Empty gastrointestinal tract weight. HKG: Weight of head, kidneys, and associated glands.

**Table 7:** Chemical composition of meat for Barki (BAR), Rahmani (RAH) and BAR × RAH sheep breeds.

Trait	Unit	Sex	BAR	RAH	BAR × RAH	*p*-Value
Moisture content	%	M	70.61 ± 1.48 ^a^	71.95 ± 2.20 ^a^	75.36 ± 1.94 ^a^	0.005
		F	69.86 ± 1.53 ^b^	72.50 ± 2.46 ^a^	73.95 ± 1.95 ^a^	0.012
		Overall	70.24 ± 1.52 ^c^	72.23 ± 2.22 ^b^	74.66 ± 1.99 ^a^	<0.001
Crude protein	%	M	20.53 ± 0.61 ^a^	21.49 ± 0.52 ^a^	21.55 ± 0.67 ^a^	0.021
		F	20.15 ± 0.78 ^b^	20.95 ± 0.67 ^ab^	21.75 ± 0.47 ^a^	0.003
		Overall	20.34 ± 0.70 ^b^	21.22 ± 0.64 ^a^	21.65 ± 0.57 ^a^	<0.001
Total lipids	%	M	3.59 ± 0.47 ^a^	3.84 ± 0.35 ^a^	2.86 ± 0.41 ^b^	0.005
		F	3.28 ± 0.54 ^a^	3.66 ± 0.24 ^a^	2.76 ± 0.39 ^b^	0.008
		Overall	3.44 ± 0.52 ^a^	3.75 ± 0.30 ^a^	2.81 ± 0.39 ^b^	<0.001
Ash content	%	M	0.99 ± 0.05 ^a^	0.91 ± 0.05 ^b^	0.88 ± 0.07 ^b^	0.014
		F	1.01 ± 0.06 ^a^	0.87 ± 0.06 ^b^	0.91 ± 0.08 ^b^	0.008
		Overall	1.00 ± 0.05 ^a^	0.89 ± 0.06 ^b^	0.89 ± 0.07 ^b^	<0.001
Collagen	%	M	2.41 ± 0.44 ^a^	2.64 ± 0.38 ^a^	2.07 ± 0.42 ^a^	0.116
		F	2.59 ± 0.42 ^a^	2.34 ± 0.35 ^a^	2.20 ± 0.26 ^a^	0.229
		Overall	2.50 ± 0.42 ^a^	2.49 ± 0.39 ^a^	2.13 ± 0.34 ^b^	0.018

Values are presented as mean ± standard deviation (SD). ^a–c^ indicate significant differences among breeds within the same row. Means sharing the same letter are not significantly different (*p* > 0.05).

**Table 8:** Gastrointestinal tract pH values for Barki (BAR), Rahmani (RAH) and BAR × RAH sheep breeds.

Trait	Unit	Sex	BAR	RAH	BAR × RAH	*p*-Value
Rumen pH	-	M	6.47 ± 0.05 ^b^	6.83 ± 0.03 ^a^	6.88 ± 0.07 ^a^	<0.001
		F	6.47 ± 0.07 ^b^	6.87 ± 0.05 ^a^	6.87 ± 0.04 ^a^	<0.001
		Overall	6.47 ± 0.06 ^c^	6.85 ± 0.05 ^b^	6.87 ± 0.05 ^a^	<0.001
Jejunum pH	-	M	6.44 ± 0.06 ^a^	6.60 ± 0.06 ^a^	6.33 ± 0.05 ^b^	<0.001
		F	6.48 ± 0.07 ^a^	6.64 ± 0.05 ^a^	6.35 ± 0.06 ^b^	<0.001
		Overall	6.46 ± 0.06 ^b^	6.62 ± 0.06 ^a^	6.34 ± 0.06 ^c^	<0.001
Caecum pH	-	M	6.38 ± 0.06 ^b^	6.80 ± 0.06 ^a^	6.71 ± 0.06 ^a^	<0.001
		F	6.40 ± 0.06 ^b^	6.82 ± 0.06 ^a^	6.71 ± 0.07 ^a^	<0.001
		Overall	6.39 ± 0.06 ^c^	6.81 ± 0.06 ^a^	6.71 ± 0.06 ^b^	<0.001

Values are presented as mean ± standard deviation (SD). ^a–c^ indicate significant differences among breeds within the same row. Means sharing the same letter are not significantly different (*p* > 0.05).

## Data Availability

The original contributions presented in this study are included in the article. Further inquiries can be directed to the corresponding authors.
